# The psychosocial barriers and enablers for managing growing up with an absent father

**DOI:** 10.4102/hsag.v30i0.2911

**Published:** 2025-09-10

**Authors:** Luyanda Mathe, Maditobane R. Lekganyane

**Affiliations:** 1 Department of Psychology, College of Human Sciences, University of South Africa, Pietermaritzburg, South Africa; 2 Department of Social Work, College of Human Sciences, University of South Africa, Pretoria, South Africa

**Keywords:** absent father, barriers, challenges, enablers, young women

## Abstract

**Background:**

This qualitative study was prompted by limited literature and knowledge around the psychosocial barriers associated with father absence among young women in South Africa and the enablers for overcoming these barriers.

**Aim:**

The aim was to explore the psychosocial barriers and enablers faced by young women because of father absence in South Africa.

**Setting:**

The setting of this study was the Central Business District of Pretoria in the city of Tshwane, South Africa.

**Methods:**

Following exploratory and descriptive qualitative research and Norman Garmezy’s resilient theory, six young women who met the predetermined inclusion criteria were recruited through purposive sampling to participate in this study. Thematic analysis strategy proposed by Braun and Clarke was used to analyse the data that were collected through semi-structured interviews. The study followed relevant ethical principles and ensured trustworthiness through the principles of credibility, dependability, transferability and confirmability.

**Results:**

The findings demonstrated that these women encounter several barriers, including emotional, financial and relationship issues, attributable to father absence from their early lives.

**Conclusion:**

Despite the various barriers encountered by these women, they demonstrated the ability to overcome them, with their resilience found to be anchored in both individual and environmental factors such as family support, a strong belief in education and self-awareness.

**Contribution:**

This study contributes to a pool of literature by adding the barriers and enablers for managing the challenges of growing up with an absent father and amplifying a call to provide psychosocial support to them and their families.

## Introduction

The phenomenon of children who grow up with absent biological fathers in their lives is a global concern (Freeks 2017, 2024). An absent father is a biological father who is not involved in his child’s life, including not physically living with them in the same household (Heartlines 2020; Sonke Gender Justice 2021). During the period 2021, South Africa had an estimated 22% of children living with absent fathers (Sonke Gender Justice 2021). Using Langa’s (2014) criteria, absent fathers in this study refer to biological fathers: (1) who are still alive but are not in contact with their children; (2) whose children do not know about them as their identity has not been revealed to them by their mothers or any adult closer to them; and (3) who are known to their children but do not acknowledge these children as theirs. Without their father’s involvement in their lives, these children often encounter challenges such as a lack of financial support, mental health-related issues, compromised well-being, poor academic achievements and feeling emotionally unloved, with the potential to negatively affect their future lives as adults (Freeks 2024; Freeks & De Jager 2023; Zulu 2014).

Despite these challenges, existing evidence points to enablers of resilience, with extended families being crucial support systems (Magqamfana & Bazana 2020; Makofane 2015; Zulu 2014). In African cultures, for instance, having an absent father does not necessarily suggest that these children are raised without male father figures to influence their lives (Swartz et al. 2015). This is aligned to the general African practice, promoted by proverbs such as ‘it takes a village to raise a child’.

### Problem statement and rationale

Fathers are important influencers on children’s development throughout their life course (Freeks & De Jager 2023; Magqamfana & Bazana 2020). Despite their crucial role in their children’s lives, South Africa has the highest proportion of absent fathers, with research reporting that one in every two fathers is absent from their children’s lives (Freeks & De Jager 2023; Ramatsetse & Ross 2022). The consequences of an absent father in the life of a child have been investigated over many years (Freeks & De Jager 2023; Makofane 2015; Ramatsetse & Ross 2022), and while the physical presence of the father alone does not necessarily ensure a positive outcome for the child or family, father absence may have detrimental consequences for families and society (Eddy, Thomson-de Boor & Mphaka 2013).

Studies have pointed to various detriments of father absence to children, including gender-related challenges, low self-perception, relationship insecurities, financial deprivation, psychological disturbances, teenage pregnancy and academic performance difficulties (Mgqamfana & Bazana 2020; Molongoana 2015). Zia, Malik and Ali (2015) investigated academic performance among adolescent girls and single mothers from diverse social and economic backgrounds who lived full-time with their fathers. Their study found that the presence of a father enhances a positive father–daughter relationship, which in turn facilitates academic achievements. For females, Schwarzwalder and Tax (2015) argue that absent fathers expose daughters to violent and unhealthy relationships and early engagement in sexual activities. Their daughters are negatively affected emotionally and experience a strong sense of abandonment and rejection from their absent fathers, which inhibits their academic performances (Freeks & De Jager 2023; Mgqamfane & Bazana 2020). In addition, the financial strain from having an absent father takes a toll on their general lives (Mgqamfane & Bazana 2020). Through this study, the researchers sought to contribute to existing literature on absent fathers, resilience and father–daughter relationships.

## Literature review

### The meaning of fatherhood

Factors such as cultural and familial ideologies are central to the enhancement of father–child relationships and therefore indispensable in the definition of fatherhood (Lamb & Tamis-LeMonda 2004). Fatherhood is commonly defined in terms of the father’s ability to care and provide for his children (Heartlines 2020). It involves having a broad range of responsibilities, including emotional development of their children, promoting inner growth and strength of their children and serving as teachers, moral guardians, protectors and providers for their children, which persist from childhood to adulthood (Tanfer & Mott 1997).

The contemporary view of fatherhood portrays fathers as having a bigger responsibility of financially supporting the child (Tanfer & Mott 1997). This change resulted in a slight shift to the role definition of parents, with mothers having more control over the domestic spheres and fathers drawn outside the home and into the workplace (Tanfer & Mott 1997). In South Africa, the system of apartheid and its policies disrupted family life, because of the migration of males from rural to urban areas, causing family separations, thus affecting the role of a father in the family (Chikovore, Makusha & Richter 2012). With the absence of a father in the family, it is mostly a daughter who becomes negatively affected. Research studies have proven that father–daughter relationships contribute to moulding sexual behaviours and curbing pregnancy, and that girls who grow up with absent fathers in their lives tend to have early sexual activities and become pregnant in their teenage years (Schwarzwalder & Tax 2015).

### Forms of fatherhood

Fatherhood is a concept that is enmeshed with societal, cultural, economic and historic issues (Bitalo, Piotrowski & Naude 2024). From a societal perspective, a father is someone who is actively involved in the affairs of his children, although how they perform such duties depends on their social backgrounds. Whereas from an economic perspective, the so-called ‘high-quality fathering’ depends on their economic contributions, culture prescribes the recognition of fathers based on practices such as paying ‘*inhlawulo* and *lobola’* (Bitalo et al. 2024:1110). Despite the various perspectives on the meaning of fatherhood, research on absent fathers is hampered by western culture and the upper socioeconomic class, from which most researchers come, which then results in a skewed perspective often without considering the fathers’ involvement in other key responsibilities (Draper & Harpending 1982). However, local studies are beginning to challenge the discourse on fathers by highlighting their several ways of involvement in caring for their children (Clowes, Ratele & Shefer 2012). A study focusing on custody, access and maintenance experiences of fathers, for instance, revealed a strong sense of family bond and the importance of the father–child relationship (Mabusela 2014). Some of the participants saw the justice system as a hindrance against strengthening the bond with their children and saw themselves as financial and emotional support providers (Mabusela 2014).

### Enablers of absent fathers

In recent years, there has been a rise in father absence from children’s lives, with literature confirming that this is because of various factors such as delayed marriage, migrant labour, violence against women and growing independence among women (Swartz et al. 2015). In the mid-twentieth century, most single-parent families arose because of the death of a spouse (Lindwall, Bailer & Daly 2011). The 1970s and the 1980 witnessed more single parent families, with the 2000s having single parents who never married (Eddy et al. 2013; Lindwall et al. 2011).

In South Africa, 31% of mothers were reported to be singlehandedly raising their children, while only 33.3% of preschool children are living with both biological parents (Freeks 2024; Statistics South Africa 2011). Among the reasons for this may be that most fathers believe that providing material and financial support defines fatherhood, a perception which often overshadows contact time as well as emotional and physical care (Swartz et al. 2015). In South Africa, where poverty is high, an emphasis on financial support may disadvantage many fathers.

### Father absence and children

There are several benefits for children with a present father in their lives, and these include the enhancement of emotional well-being (Freeks 2024; Langa 2014). Whereas fathers often engage in activities such as physical play to promote the child’s intellectual skills and social development, mothers use verbal expressions and teachings to promote these skills (Teitelbaum 2013). Therefore, this suggests that the absence of a father leaves a vacuum in the child’s intellectual and social development.

Absent fathers contribute to the psychosocial stress of their children as well as the unresolved paternal identity issues that might potentially have negative consequences on children (Freeks 2024).

Absent fathers are associated with negative effects on children’s social and economic well-being. This connection has been illustrated through a relationship between father absence and children’s delinquent behaviour, poor intellectual functioning and poor well-being (Mavungu 2013). A child who has an absent father tend to experience loss, confusion and humiliation (Chikovore et al. 2012). Acknowledgement by the father is an important part of identity development in children particularly in South Africa, where children take their father’s clan name if they are acknowledged (Chikovore et al. 2012). Thus, for many children, having an absent father does not only affect them financially, but it also affects their identity.

### Absent fathers and a girl child

It is assumed that fathering a daughter can be challenging, especially for men, because daughters are closer to their mothers (Zia et al. 2015). Most fathers often doubt their importance to their daughters compared to their sons (Nielsen 2006). The reality is that their involvement in their daughters’ lives is just as important as that of the mother, because they also play a role in their psychological health, self-confidence, school achievement and intimate relationships (Zia et al. 2015). For many girls, having an absent father leads to complexities in intimate relationships, depression and poor self-esteem (Magqamfana & Bazana 2020; Ramatsetse & Ross 2022). Furthermore, fathers generally have a greater impact on the lives of their daughters, particularly the level of happiness, trust and quality of relationship with opposite genders. Daughters who have a good relationship with their fathers tend to develop confidence, a sense of independence and become successful in both academic and career activities (Nielsen 2006).

### Absent fathers: A resilient theoretical perspective

A good research study is based on a good theoretical framework. By theoretical framework, Grant and Osanloo (2014) refer to the basis upon which knowledge is constructed for research purposes. A theoretical framework underpins the study’s literature review, methodology, rationale, problem statement, goal and the questions (Grant & Osanloo 2014). It guides the research topic, including the selection of concepts and definitions that are relevant (Grant & Osanloo 2014).

Norman Garmezy’s theory of resilience was adopted to provide a structured approach to understanding how individuals develop coping mechanisms in adverse situations such as young women who grow up with absent fathers. The study effectively applied the concept of protective factors at individual, familial and community level to interpret its findings. These protective factors include *individual factors* such as easy temperaments in children and a positive outlook in adults, *familial factors* such as the support from grandparents, social fathers and siblings and, lastly, *supportive factors* such as the community at large, including neighbours and teachers (Shean 2015). Using the theoretical framework for this study as the guide, it was essential to know both the participants’ resilience and adversities. By adopting Norman Garmezy’s theory of resilience, the researchers sought to focus on specific principles such as ‘protective factors’ and ‘adverse events’, thereby allowing them to explore the participants’ experiences in a more guided and in-depth manner in the study. In South Africa, Malindi, Theron and Theron (2013) found that youth had protective factors such as an active community-based support system operating in their lives and a value-driven personality, which positively influenced behaviour. This showed that resilience was the result of a combination of various internal and external factors.

### The study aim and questions

This study sought to explore the psychosocial barriers and enablers faced by young women who grew up with absent fathers in South Africa.

From this aim, researchers proceeded to formulate the following questions:

- *What are the psychosocial barriers faced by young women because of father absence in South Africa*?
- *What are the enablers for young women to overcome the psychosocial barriers associated with father absence in South Africa*?

## Research method and design

This study was grounded in exploratory qualitative research. A qualitative study enables an understanding of complex phenomena such as the experiences of the participants (Hall & Liebenberg 2024). In qualitative research, data collection is conducted through methods such as interviews, focus group discussions, observations or analysis of records, reports, photos and documents, with the aim of capturing specific events of the study (Hall & Liebenberg 2024). A sample of six black women aged between 21 and 30 years was purposefully chosen for the study. To participate in this study, participants had to (1) be females aged between 21 and 30 years; (2) have experience of growing up with an absent father; and (3) identify themselves as black women. For data collection, individual semi-structured interviews were utilised as supported by an interview guide containing a set of six questions focusing on the participants’ experiences pertaining to leading life without the involvement of a father. The interviews that were conducted face-to-face in English language, were thematically analysed according to Braun and Clarke’s (2006) framework to generate four themes and eight subthemes.

To enhance the study’s rigour, the principles of credibility, confirmability, transferability and dependability were applied (Ahmed 2024; Ghafouri & Ofoghi 2016). For *credibility*, extended and persistent involvement were maintained and involved reading the transcripts a few times to reflect on the process of research (Ahmed 2024; Ghafouri & Ofoghi 2016). *Transferability* and *dependability* were maintained by means of thick description, which involved detailing all the processes, including the methodological process followed (Ahmed 2024; Ghafouri & Ofoghi 2016). They (transferability and dependability) also involved reporting the findings through direct verbatim quotes from the participants. All these sought to enable other researchers to apply the study’s findings to contexts similar to their study site. For *confirmability*, reflexivity, member-checks and peer debriefing were adopted (Ahmed 2024; Ghafouri & Ofoghi 2016). Through reflexivity, researchers were able to reflect on their influence on the research process and notice that being young Africans, they assumed that they knew some of the experiences of the participants and ended up not probing some of the crucial areas during the interviews. This assumption costed them because they ended up with data that was not rich enough. By using member-checks, researchers afforded participants an opportunity to verify if indeed the collected data reflected what they told the researchers (Ahmed 2024; Ghafouri & Ofoghi 2016).

### Ethical considerations

In conducting this study, researchers were mindful of the significance of the ethical principles of research. In that regard, they subjected a research proposal to the Institutional Review Committee of the University of South Africa for ethical protocol and approval. The committee was satisfied that the study met acceptable ethical standards and approved the protocol on 2016/03/09 with ethical clearance number (Ref no: PERC-16011). Informed consent was upheld by ensuring that all participants were properly informed about the study purpose and by signing an informed consent form before they could participate. The study also involved the protection of participants’ data and their identities as required by confidentiality and anonymity. Instead of using the participants’ real names, numbers were used to label them. Reporting of the study’s findings was also done with dignity to reflect the participants’ views as expressed during the interviews. The collected data will be stored for a period of 15 years as required by the policy of the university of South Africa on research. To guard against the emotional repercussions of the study on the participants, a clinical psychologist was organised to render debriefing after the study, though there were no incidents that required her services. Generally, the study upheld beneficence and nonmaleficence, which enabled researchers to refrain from harming the participants and ensuring that the study ultimately benefits the participants (Forzano & Gravetter 2012).

## Results and discussion

### Participants’ demographic profiles

The participants were assigned numbers to protect their identities and promote ethical principle of anonymity. Six participants, who were all young black women, took part in this study. Although they were from several parts of the country, they were all based in Pretoria, South Africa’s City of Tshwane, for educational or employment purposes. The following section outlines the demographic profiles of each participant.

### Participant one

Participant one was 30 years old. She was a Xhosa girl from the Eastern Cape and only knew isiXhosa, isiZulu and English languages. For the purpose of the study interviews, she elected to communicate in English. Her highest qualification was a Diploma in Events Management, and her occupation was as an events coordinator at a company based in Pretoria. Her presence in Pretoria was only for employment purposes. According to participant one, she was told that her father never acknowledged paternity, and she only heard that he is somewhere around the Eastern Cape. Her mother was a single woman with three children, and participant one was the first child. Her other siblings shared a father who was present, even though not staying with their mother full-time. Participant one was raised by her maternal grandmother, with her uncle playing the role of a father figure. Participant one did not have any reunification with her father because she never met him, nor did she know anyone from the paternal side of her family.

### Participant two

Participant two was 27 years old, originally from Limpopo province in South Africa. She was in Pretoria for tertiary educational purposes. At the time of this study, participant three was in her second year of tertiary studies for a Diploma in Public Relations. Although her home language was Sepedi, participant two reported that she was a Swati by birth and therefore could speak Sepedi, siSwati and English. However, she was articulate in English during the interview. According to participant two, she also had an absent father. Her father had two wives, and her mother was the first wife. The other wife had her own house and children, so her father lived with such a wife and never had time for participant two and her mother. He would only visit them for about 30 min and then leave, and sometimes she would not see him at all. Participant two’s only sources of support were her mother, uncles and aunts from the maternal family side. She was the sixth child from a family of six. As far as participant two can remember, she only started to develop some kind of relationship with her father when she was in high school at around 18 years.

### Participant three

Participant three was 21 years old, and she was a full-time student doing her fourth year of tertiary studies in Bachelor of Education. She was a Xhosa tribe from the Eastern Cape, and her home language was isiXhosa. However, she was clear and articulate in English and isiZulu. For the purpose of the interviews, she preferred to use English. She was in Pretoria for study purposes. Participant three was a fourth child from a family of four. All she knew was that her parents got divorced when she was only two. She only knew her father as a man with substance abuse-related issues, who was paying maintenance and who would sometimes come home even though he was not welcome. Participant three’s main sources of support were her mother, her siblings and friends. Besides the financial support in a form of maintenance paid by her father, participant three’s father and paternal family members were not providing any other support. At the time of this study, participant three’s father was still absent and did not have any relationship with her.

### Participant four

Participant four was a Zulu woman who originally came from the KwaZulu-Natal province. Her presence in Pretoria was for employment purposes. Her highest qualification was a Diploma in Business Administration. Participant four was 30 years old. She was working as a personal assistant at a private company based in Pretoria. Although her home language is isiXhosa, she was clear and articulate in English, which is the language she preferred to use during the interview. According to participant four, her mother was in a relationship with a married man, and their relationship ended up with her birth. She never met her father. Participant four did not worry much about her father’s presence until she was in high school. Her father was only financially available to her. Her primary source of support was her mother, maternal grandmother and uncle; her father and the maternal family were never involved in her life. Participant four never got to reunite with her father, and she does not hope that such will ever happen.

### Participant five

Participant five was a 23 years old woman from the Northwest province of South Africa. She was not only a Motswana speaker but also articulate in English and Afrikaans and slightly isiZulu. Like other participants, she also preferred to be interviewed in English. Her presence in Pretoria was purely for tertiary educational purposes. She was doing her third year of tertiary education in Bachelor of Education. Participant five’s reasons for growing up with an absent father were because of separation. Her father used to be present in her life until he decided to leave permanently without telling her mother the reasons. He also stopped supporting her, including financially. According to participant five, she had aunts (her mother’s sisters), who were supportive to her. At the time of the interview, she still did not have any bond with her father, whom she said was the one who walked away. Participant five and her father did not reunite.

### Participant six

Participant six was 27 years old. She was working as a research intern in Pretoria. The main reason for her to be in Pretoria was employment. Participant six’s highest qualification was a Diploma in Office Administration. She was a Zulu woman from KwaZulu-Natal province of South Africa and her home language was isiZulu. She was, however, articulate in English, isiXhosa and siSwati. For the purpose of the study interviews, she preferred to speak in English. Regarding the involvement of her father in her life, participant six said he was not always present because he was working out of the province. Although her father would be home over the weekends and was financially supportive, he was not really involved in her upbringing. Her primary source of support was her mother, maternal grandparents and aunts. According to Participant 6, no efforts were made to restore her relationship with her father.

## Findings on themes and subthemes

Four main themes that originated from data analysis are presented as follows.

### Theme 1: Participants’ views on absent father and the reasons for their absence

The first theme that emerged from the analysis process involved the interpretations of the participants regarding the meaning of an absent father and the reasons for fathers to be absent. In this regard, two subthemes were generated, namely: a lack of emotional and financial support by a father and the reasons for father absence in the life of a child.

#### Subtheme 1.1: A lack of emotional and financial support by a father

From the participants’ perspectives, absent fathers were described in different ways, such as not contributing financially as well as being emotionally absent. These aspects were clustered into emotional and financial absence in fatherhood.

This is what participant one had to say about the physical and emotional aspects of an absent father:

‘[*U*]hm absent, I understand that it is a parent that is not present physically, emotionally and financially.’ (Participant 1, 23 years old, Student)

Participant three described an absent father in terms of the psychosocial absence as well as the economic absence. He had this to say:

[*W*]ell absent father, is absenteeism, not being there at all! Absent in terms of emotions and there is no connection, financially absent, not getting any support there. Someone who is not there in the space …’ (Participant 3, 21 years old, Student)

Another participant who pointed to the psychosocial and economical aspects of absence was participant four, who told researchers as follows:

‘I would say yoh it is a lot, I would say it is a father that is not present … in all ways, it could be an absent father that is married to the mother, you know. Absent, I understand that it is different ways; financially, emotionally, psychologically.’ (Participant 4, 30 years old, Personal Assistant)

Unlike other participants, participant two was not really bothered by the financial aspects of father presence. She was more bothered by the lack of emotional support. She explained her views as follows:

‘An absent father in my own words I would say it is somebody who can either be there financially, but not be there emotionally in sense of they only feed money, money, money. Whether it is rich money or not, when I say rich money, I mean like they can just buy you.’ (Participant 2, 27 years old, Student)

The participants indicated various ways in which a father can be absent, which included emotional, physical, financial and psychological reasons. Although some participants received financial support from their fathers (i.e. Participants Two and Four), they still considered their fathers to be absent, given their interpretation of a present father as a father who is also emotionally present. This seems to contradict some parts of the existing literature, which reveals that there is great emphasis on financial support in certain cultures. Fathers are not considered adequate unless they have financial means to support their children, and they also consider themselves providers based on the material or financial support to their children (Eddy et al. 2013). From the participants’ narratives, it is clear that financial support alone is not enough to qualify one as a good father. This is a perspective corroborating Swartz et al.’s (2015) assertion that an emphasis on financial support should not overshadow other important aspects of fatherhood such as emotional care, physical care and contact time. For Mavungu (2013) and Eddy et al. (2013), financial support is the most important contribution a parent can provide to their child. Mavungu (2013) cautioned against this narrative, arguing that fathers are mainly seen as providers and that this often precludes alternative fatherhood roles.

#### Subtheme 1.2: Absent fathers as a norm in the communities

Another view expressed by the participants regarding absent fathers was that it is generally common for children to grow up with absent fathers in their communities. These perspectives were reported under subtheme 2 as outlined next.

The first participant to express the views regarding absent fathers being a norm was participant one, who had this to say:

‘In the community, it’s very normal, it is very normal for most of us. In, where I lived, we didn’t have fathers. It was very rare that you had friends that had both their parents or had involved fathers. So it was a normal thing; it wasn’t something that is out of the ordinary.’ (Participant 1, 23 years old, Student)

In another interview, participant three indicated that the absence of fathers has been normalised. This is what she told researchers:

‘I think it is not seen as something big, it is normalised, people have become desensitised to it now, so it is my family and that one, it is not seen as a big deal, most of my friends and the community, they have single mothers or females in that community of house.’ (Participant 3, 21 years old, Student)

Participant four explained how her friends also grew up with absent fathers:

‘It happened so often it does not really mean anything, all my friends from the neighbourhood I grew up in did not have dads, there was one only [*name*], she stood out because she had a single dad, yeah her mom was absent.’ (Participant 4, 30 years old, Personal Assistant)

For participant five, in her community, this normalisation of absent fathers took a form of blaming the mother. This is how she explained it:

‘[*W*]ith my mother it was like “why did she have to fall pregnant,” she thought she could trap him.’ (Participant 5, 23 years old, Student)

As reported by participant six, normalisation of absent fathers took the following form:

In the community I would not say it is something that really bothers most of the families, it was something normal, because in some of the families, it was that the father needed to take care of the family, that is why he is not there, he is taking care of the family, and you should be grateful for that. (Participant 6, 27 years old, Admin Officer Intern)

The narratives outlined above in relation to the normalisation of absent fathers in the communities attest to a picture of most South African communities. Freeks (2017) argues that South Africa is swiftly normalising fatherlessness, and with this come various challenges such as disintegrated families, and problems that are financial and social in nature, including poverty and child behavioural issues. Approximately 50% of South African fathers lack daily communication with their children and black fathers have been found to be the highest when it comes to father absence (Chikovore et al. 2012). Although participants did not point out the reasons for the normalisation of absent fathers in communities, Molongoana (2015) argues that in South Africa, fathers often leave their homes to find jobs in the city so that they can support their families. In some situations, father absence is justified by factors that are beyond human control, including unemployment, poverty and income inequality, which suggests that biological fathers may not adequately contribute to raising their children as they could not afford to do so financially (Clowes et al. 2012).

An interpretation of the current findings from Garmezy’s resilient theory suggests that as much as father absence is a community concern, it also resembles external support factors from outside the family (Shean 2015; Wang, Zhang & Zimmerman 2015). Despite the widely known negativity associated with it, its normalisation in the community implies that children who grow up with absent fathers become less exposed to stigmatisation and related issues because people are used to the phenomenon. It may also attract efforts to ensure that these children receive the necessary support.

### Theme 2: The experiences of young women in the absence of their fathers

From the analysis report, participants had both benefits and challenges associated with absent fathers, and they are presented as follows:

#### Subtheme 2.1: The benefits associated with having an absent father

Participant 6 reported how the absence of her father enabled her to be more careful in other relationships as well as to be independent:

‘Like, it influenced me and not my decisions, just other things you know. Okay, I have learned to be independent and not to wait uhm firstly not to wait for opportunities to come to me. Of course, to look for the opportunities and secondly and my mother used to say, there is nothing you cannot do with cents, as a person you can do anything, despite that you have a father or not, as a person you can do extremely well without even knowing your father.’ (Participant 6, 27 years old, Admin Officer Intern)

Participants two and three further mentioned that the father’s absence in the household also had some positive effects on the family. This is what participant 2 stated:

‘I think it made us close as a family. Now, in our minds, it is only us … us against the world, well against whatever … it made us to be the tight knit that it is only us … and we are only the people who can support us whether is it financially or emotionally we are there. I can see it now, as we are adults when this one is going through this, we are all there.’ (Participant 2, 27 years old, Student)

Our interview with participant three also pointed to a similar sentiment:

‘I think in a sense that we had to rely on each other a lot, so we had to say, Guys this is what we have, and we have to work with it to our best. It shows because we are now in our 20s and in different stages in our lives, my brother is getting married. My sister just started her job, and my other sister just had a baby, so these are stages where we really need each other.’ (Participant 3, 21 years old, Student)

From the assertions made by the participants, it appears that having an absent father also had some benefits. However, research on absent fathers hardly explores whether there are any benefits of having an absent father. For example, although very few, the findings suggest that there are positive individual characteristics that one develops because of having an absent father. Our findings support this perspective by suggesting that absent fathers also enable a sense of independence among some of the children later in their lives. This is an attestation of the individual protective factors associated with Norman Garmezy’s resilient theory (Shean 2015). Reports such as a sense of independence that had developed from experiences of having an absent father by participant six, for instance, indicate the improvement of cognitive skills, which are the individual protective factors. People like participant two, who pointed to family unity, and participant three, who referred to reliance on each other (interdependence) as a family, reflected the familial protective factors of support through a sense of cohesion and warmth (Shean 2015). The findings also reflected Ramatsetse and Ross’ (2022) findings, wherein participants reported the advantages of growing up without a father such as being strong and independent. However, in the same study, and in confirming Wang et al.’s (2015) argument that resilience differs per individual, some participants reported the benefits of not having a present father.

#### Subtheme 2.2: The challenges of young women with an absent father

Regarding the challenges of the participants in relation to absent fathers, they pointed to their childhood challenges as well as those that they encountered later in their adulthoods. These included emotional problems such as anger issues and feelings of rejection, relationship problems with the opposite sex and financial problems.

Participant two explained her challenges as follows:

‘Huh, well other than … fighting … I spent all my life fighting for what I needed, what I want. It did not come out how I would have loved if he were there …’ (Participant 2, 27 years old, Student)

Another participant who reported the challenges associated with absent fathers was participant three, who explained her situation by saying:

‘I have faced challenges, the biggest was to accept that there are people with dads, and I did not have a dad and it is not that there was something wrong with me so that made me stronger. It was a challenge to not be bitter to people who have dads and be accepting of them.’ (Participant 3, 21 years old, Student)

Participant three added:

‘I think I did not know the circumstances but looking back I see that my mom struggled a lot financially with four kids and juggling work with four kids, I am only seeing this now, I was not aware of it. I see she was struggling if the father was there maybe things would have been easier …’ (Participant 3, 21 years old, Student)

In the case of participant four, she had to deal with disappointments such as failed relationships. She had this to say:

‘Oh, it teaches you not to have very high expectations for people and things like that, leave room for disappointment, it is not really like a negative thing, but it is a way to kind of deal with uhm failed relationships and things like that and sometimes it makes it worse though because you are like ‘this is happening and now it is happening again’ … also to know when somebody is absent in a relationship with you.’ (Participant 4, 30 years old, Personal Assistant)

Participant six also alluded to the challenges of relationships with males:

‘When I interact with males, I look for a trait that related to my father, at some point I also want to say I don’t want those traits and things that are like my father. Maybe this person would behave exactly like my father and sometimes there is that trait, and sometimes I take it as it is and continue with life …’ (Participant 6, 27 years old, Admin Officer Intern)

The findings of this study regarding participants’ challenges in relation to having an absent father corroborate the reviewed literature around the challenges associated with absent fathers (Freeks & De Jager 2023). The challenges, as reported by the participants, demonstrated that fathers have a significant impact on their daughters, including their ability to appreciate, trust and relate well to other males (Freeks & De Jager 2023; La Guardia, Lertora & Nelson 2014; Zia et al. 2015). Father–daughter relationships serve as a model for how females learn to interact with and accepted by males (La Guardia et al. 2014). Although lack of a healthy father–daughter relationship can lead to insecurity with males, as shown by the findings of the study, this does not mean these young women will be unable to navigate successfully through their life journey. From a resilient theory, individuals do experience adversity in their lives but also develop positive adaptations by overcoming them. The fact that these young women had challenges associated with father absence does not necessarily mean they will not succeed in their lives.

### Theme 3: The current state of relationships between the participants and their absent fathers

One of the things that emerged from the findings was the status of the participants’ relationships with their fathers. Some of the participants reported an enduring negative relationship with their fathers. These experiences were filtered to generate two subthemes that are presented as follows:

#### Subtheme 3.1: Lasting disappointment

Participant three reported the saddening end, with the death of her father. She had this to say:

‘He passed away, but I think he would have done what he does best; raise my hopes and then leave me there.’ (Participant 3, 21 years old, Student)

For participant three and her father, the relationship became so sour to an extent that they never spoke for a period of a year. This was shared by participant three:

‘I have not spoken to him since last year (2016), December. There was a ceremony back at home and I went there, he asked my mother if I could come so I went there and I saw him. I am very close with my uncle, my dad’s brother, he is older. My dad is the youngest in the family just like me.’ (Participant 3, 21 years old, Student)

Similar sentiments reported by participant three were also shared by participant one, who had not spoken to her father for two years:

‘I think we haven’t talked in like 2 years or something, he tried to SMS me beginning of this year and I blocked him …’ (Participant 1, 23 years old, Student)

Consistent with Norman’s resilient theory on the existence of adversities, participants reported that their relationships with their fathers ended up being so sour to an extent that they stopped talking for a maximum of 2 years. These kinds of issues seem to have support in some existing literature, with studies such as Pyper, De Klerk and Spies’ (2015) revealing that these young women develop a lack of trust towards their fathers and that some resolve not to involve them in their lives. However, in attesting to the resilient theoretical perspective, which argues that the adverse situations of the participants can always have a positive side, existing evidence shows that women who grow up with an absent father tend to be resiliently strong, victorious, assertive, self-reliant and empowered because of the support that they receive from extended family members (Magqamfana & Bazana 2020). This sense of strength, self-reliance and assertiveness is once again proven by the participant who decided to close their chapter of father absence and continued to live their lives without these fathers.

#### Subtheme 3.2: reuniting with their fathers

Another subtheme that emerged from data analysis was that participants managed to eventually reunite with their fathers.

Participant six was among those who reunited with her father, and she had this to say:

‘My father likes me now, you know, he likes me a lot. I think the mistakes he did when we were young because I think it was 2003 or 4, or maybe 2010 or 2011, he came and set us down, it was I, my older sister, and my two older brothers, at the time things were falling apart at home.’ (Participant 6, 27 years old, Admin Officer Intern)

Participant four also reported a positive relationship with her father. This is what she said:

‘We are very good now, at the moment, we have a very friendly relationship, we talk about politics, he calls me, and we talk about what happened on whatever show and if you listened to Power FM on the radio and sometimes, I feel like we are trying to make up for lost time.’ (Participant 4, 30 years old, personal Assistant)

What is reported by the participants is central to resilience, which, as defined by Garmezy, entails the ability to recover and maintain adaptive behaviour after a stressful experience (Shean 2015). From the participants’ narratives, it is clear that out of the pain of navigating through their lives with an absent father, they still recovered and even had guts to reunite with them. Their assertions in this regard find support in studies such as Pyper et al.’s (2015) South African study of the experiences of young adult women regarding the emotionally absent fathers, which revealed that young women resolved to keep peace by reconciling with their fathers. Another South African study by Ramatsetse and Ross (2022) investigated the psychosocial impact of an absent father during childhood and adolescence stage of daughters on their adulthood as women. However, unlike our study regarding this subtheme, their findings were mixed, with some participants reporting hatred towards their fathers, others reporting no feelings, while some expressed no hatred at all even though their fathers were uninvolved in their lives (Ramatsetse & Ross 2022).

### Theme 4: Overcoming the challenges associated with absent fathers

Alongside the challenges reported by the participants were also protective factors that enabled them to overcome their challenges. Although they have experienced challenges while growing up because of having an absent father, participants also felt that they have been able to face these challenges head on. Their responses were clustered into two types of protective factors, namely, individual resilience and familial support:

#### Subtheme 4.1: Individual resilience

One of the forms of resilience as reported by the participants was what researchers considered to be individual resilience. As demonstrated next, participants explained how they were able to draw from within themselves various strategies to assist them in managing the issues faced in relation to absent fathers.

One of the participants who attested to this was participant two, who had this to say:

‘I would say that the whole forgiving thing, forgiving my father and deciding that this is what I want with my life. I want to be a successful woman and success to me would not just be money. Even being able to hold my emotions, being able to take care of myself, to provide for myself. I can go get myself a job and provide for myself and that is for me overcoming the challenges …’ (Participant 2, 27 years old, Student)

In the case of participant four, she had this to say:

‘Now when I have a good day, I just go with it, I am so appreciative of good days so much, you do not understand how much I appreciate a good day … That is when I decided that okay, I need to start getting healthy in every way again and I have told myself that I would definitely not go back home because I am going to continue staying in Gauteng and just make my midyear career dreams come true because this is the best place for me …’ (Participant 4, 30 years old, Personal Assistant)

For participant five, their resilience took a form of self-motivation to always wish to do more. This is what she told researchers:

‘I was able to push myself to do more. As much as the bad things, the bad, bad horrible things, at some point it did bother me. I was like ‘girl, if you do not want to end up like that man, if you do not want to have kids who feel the way that you feel about your father, don’t, you have to pick yourself up’ towards a positive way.’ (Participant 5, 23 years old, Student)

Participant one reported that she had to seek therapy in order for her to forgive her father for what he had done. In this regard, participant one had this to say:

‘Also, to be honest, being in therapy has also helped me to overcome my challenges of having an absent father or to address my issues that I have with him. I am very active in my therapy sessions, we don’t talk about him much, but we have talked about him in depth in order for me to forgive him. So being in therapy helped me overcome that.’ (Participant 1, 23 years old, Student)

In the case of participant six, she reported that what assisted her was to be resilient by continuing with her life and accepting her situation of having an absent father:

‘My mother would say, if you are a man or a woman, at the end of the day get over it, do house chores. So, for me being like this is not because my father was there or not there.’ (Participant 6, 27 years old, Admin Officer Intern)

Unlike her other co-participants, participant three indicated that his refuge was religion. She had this to say:

‘Religion played a big role, church played a role and God because I had to be very forgiving and learn forgiving and it does not come easy. I had to remind myself of this in my prayer and God helped …’ (Participant 3, 21 years old, Student)

Among the strategies adopted by the participants in managing their challenges was to simply draw from within themselves. As revealed by the participants, strategies such as forgiving, heeding to God, looking after themselves from a health point of view and focusing on the positive aspects of life were essential. Authors such as Al-Mabuk, Cardis and Enright (1995) encourage forgiveness as they believe that the person who chooses to forgive overcomes negative effects such as resentment and cognitions like harsh judgements, and substitutes more positive affect and cognition towards the person they are forgiving. Furthermore, Al-Mabuk et al. (1995) reported that there are physiological and psychological benefits to forgiveness, and they include a subsided heart rate, relaxed breathing, reduction in anxiety, hostility, depression and anger.

Research suggests that forgiveness can be healthy and restorative (Al-Mabuk et al. 1995). There was also a link between forgiveness and religion, as reported by Participant 3, who reported that religion played a role in her learning to forgive. Individual factors such as positive emotions, self-enhancement and repressive coping foster resilience in a person (Magqamfana & Bazana 2020; Ramatsetse & Ross 2022). Repressive coping involves an interpretive bias of negative information (Furnham & Lay 2016). Through regressive coping, participant two, for instance, was able to forgive her father, while participant five decided to pick herself up and focus on the positive things. Similar findings were reported by a South African study of the subjective experiences of black women growing up with an absent father, where young women elected to focus on the positive side of their experiences as opposed to their absent fathers (Kamau & Davies 2018). Unfortunately, and contrary to our participants, those who took part in Ramatsetse and Ross (2022) adopted destructive individual coping strategies such as smoking and drinking liquor, hoping that they will manage their negative emotions. This subtheme demonstrated the essence of Norman Garmezy’s resilient theory. From Garmezy’s resilient theoretical view, it is evident that a sense of individual factors played a crucial role in participant two who developed a spirit of forgiveness, participant four who appreciated the good days and participant five who pushed herself to do more (Shean 2015). Familial factors became instrumental in participant six through her mother’s courage, while external factors became evident in participant three who sought refuge in religion.

#### Subtheme 4.2: Familial protective factors

Alongside the individual resilience was the familial protective factors, as reported by several participants below: Participant one, for instance, had this to say about familial protective factors that assisted her in managing the challenges of living life with an absent father:

‘The family did have a big role in me overcoming my challenges … also being close with my siblings, my brothers that I just met, having them around has also helped.’ (Participant 1, 23 years old, Student)

For participant two, familial sources of support were her mother and sister. This is what she had to say to the researchers:

‘My mom and my older sister, those were the proper people who helped me, particularly when my brother started working, because my brother is much older than I am, I think like 8 years older than I do.’ (Participant 2, 27 years old, Student)

Similar sentiments shared by participant two were also reported by participant three, who received support from her family. This is what she said:

‘It was my siblings and my mother. They were the most involved and of course my friends as well. I actually got close with my friends and my friends know about me more than my family. I shared that open space with them and my mom.’ (Participant 3, 21 years old, Student)

In the case of participant four, her grandparents were instrumental sources of support. She explained her situation as follows:

‘My relationship with my grandparents was just amazing. I am very close with my grandmother; we have very similar personalities compared to my mom. She is a warm person, she likes people.’ (Participant 4, 30 years old, Personal Assistant)

Studies on resilience show that despite exposure to environmental and familial deficits, a significant number of individuals adapt to such stressors by adopting positive behavioural patterns, which lead to favourable outcomes (Garmezy 1991; Magqamfana & Bazana 2020; Makofane 2015). What is outlined by the participants in relation to the familial protective factors demonstrated Norman Garmezy’s resilient theory. From this theoretical perspective, social fathers such as uncles, male cousins and grandparents play a crucial role of parenting and children are socialised to accept them as parents (Makofane 2015). The important role that family has played in participants’ lives has undoubtedly assisted them in being resilient. In supporting the current findings, literature demonstrates that raising children in South Africa often involves grandparents and other extended family members as caregivers (Sonke Gender Justice 2021; Magqamfana & Bazana 2020; Zulu 2014). The role played by grandparents is often an enriching one, as they can teach their children traditional values and guide them. They also see this as a second chance for improving their own parenting skills (Fuhri 2013). Whereas maternal support is constant and dependable in single-mother households, having a social father is beneficial for a child’s well-being (Makofane 2015). It was, therefore, not surprising for people such as participant one to be appreciative of the role played by her elder brother who just started working, as well as participant two who recently met her supportive brothers. Reading this from Norman Garmezy’s theory, the entire subtheme is entrenched in the familial factors. The role of siblings, mothers, aunts and grandparents became sources of strengths for participants during their difficult times.

### Study’s limitations

Although the study is undoubtedly informative, it was conducted with a smaller sample. The site from which the study was conducted was also small, thereby limiting the potential diversity of experiences and perspectives. Interpretation and application of the findings should, therefore, be done cautiously in view of these limitations.

## Conclusion

With the purpose of exploring the psychosocial barriers and enablers faced by women because of father absence in South Africa, this study used Norman Garmezy’s theory of resilience. This study has demonstrated that resilience is about reflecting on the capacity for recovery and maintaining adaptive behaviour when undergoing a stressful event (Garmezy 1991). Being resilient does not imply immunity to negative life events, as is apparent in the findings of this study. Although this study demonstrated that father absence is a challenging experience for young women, particularly in emotional and financial terms, it was also a general norm in their communities for children to be raised in the absence of their fathers. As a result of these experiences, some young women found themselves having lasting disappointments, while others found ways of reuniting with their fathers. As held by Norman Garmezy’s resilient theory, the study demonstrated that despite the challenges associated with father absence as experienced by young women, they tend to manage through the individual and familial support factors such as getting over it, pushing themselves to do more, receiving care and support from siblings, grandparents and other family members in order to cope.

With the above findings in mind, psychosocial support programmes have proven to be effective in supporting coping strategies adopted by people with challenges such as growing up with absent fathers. It is, therefore, essential to design, develop and implement psychosocial support programmes that focus on supporting young women with absent fathers in order to strengthen their coping strategies. Although this study has contributed to addressing limited knowledge and literature around the subject of young women and absent fathers, it is essential to conduct further studies on a broader scale, focusing on participants from diverse population groups, including an exploration of the perspectives of absent fathers themselves regarding young women who grow up without them. This will enrich existing literature and provide various dimensions from which the subject of fatherhood and father absence can be understood.
